# Evaluation of Artificial Intelligence's Ability to Explore Literature on Orthopedic Navigation and Related Surgical Anatomy

**DOI:** 10.7759/cureus.94768

**Published:** 2025-10-17

**Authors:** Dimitrios Chytas, Angelo V Vasiliadis, Ethan Choucroun, Tanisha Naresh Chindore, Taha Ouhenach, Derin Eva Sadiq, Michael-Alexander Malahias

**Affiliations:** 1 Physiotherapy, University of Peloponnese, Sparta, GRC; 2 Sports Trauma and Orthopaedics, St. Luke’s Hospital, Thessaloniki, GRC; 3 School of Medicine, European University of Cyprus, Frankfurt, DEU; 4 Orthopaedics and Traumatology, Clinica Ars Medica, Gravesano, CHE

**Keywords:** artificial intelligence, augmented reality, chatgpt, large language models, orthopedics, scholargpt, surgical anatomy

## Abstract

Introduction

Artificial intelligence has recently garnered increased research interest in orthopedics; yet, its role in the exploration of orthopedic literature remains unknown. We aimed to evaluate the ability of ChatGPT and ScholarGPT to identify and outline literature on orthopedic navigation and the visualization of related surgical anatomy.

Methods

We asked ChatGPT and ScholarGPT to list and summarize five studies: 1) about augmented reality-based navigation in orthopedic surgery, 2) about how well augmented reality-based navigation enabled anatomical accuracy in orthopedic surgery, and 3) which compared augmented reality-based navigation with another navigation technique in orthopedic surgery. Regarding each query, we evaluated how many studies were correctly detected and accurately summarized.

Results

ChatGPT scored excellently in identifying studies across all three queries. However, its performances in accurately summarizing these studies were 40%, 60%, and 60% respectively. On the other hand, ScholarGPT’s performances in identifying were 60%, 40%, and 0%, respectively, while in summarization, they were 0%, 20%, and 0%, respectively. Both platforms exhibited bias in favor of augmented reality-based navigation across all queries.

Conclusion

ChatGPT and ScholarGPT are not yet able to provide researchers with reliable data from the literature on orthopedic navigation and related surgical anatomy. Ongoing artificial intelligence development may essentially reinforce these platforms’ potential to play a more significant role in orthopedic research.

## Introduction

Among modern technological orthopedic advances, artificial intelligence (AI) has recently garnered increased research interest. Misir showed that AI can significantly contribute to orthopedic trauma care by performing fracture detection and classification, enhancing diagnostic accuracy, enabling optimal treatment selection, and identifying high-risk patients for specified interventions [1]. In terms of reconstructive surgery, it has been shown that AI can improve preoperative planning, facilitate intraoperative navigation, and enhance the anticipation of postoperative complications [2]. Apart from clinical practice, AI can also essentially contribute to orthopedic education [3]. Das et al. found that AI can give objective feedback, offer adaptive learning modules, and enable skill acquisition in risk-free environments, thus supplementing traditional training methods [3]. However, AI needs further development to play a more significant role in orthopedics [1-3].

Among AI platforms, ChatGPT has notably attracted the interest of orthopedic researchers. The review by Zhang et al. demonstrated that there is remarkable heterogeneity in the literature regarding ChatGPT’s accuracy in diagnostic tasks, disease classification tasks, and answering questions in orthopedic examinations [4]. Morya et al. showed that ChatGPT may contribute to preoperative planning, surgical techniques, and patient education, yet presents limitations, such as biased responses, which demand cautious use [5]. 

ScholarGPT, a version of ChatGPT specialized in academic research, has been minimally explored, while there is a lack of studies to investigate its potential in orthopedics. Baris and Baris found that ScholarGPT outperformed ChatGPT in answering questions about a surgical subfield [6], while Chen et al. showed ScholarGPT’s performance to be poor in identifying and selecting relevant papers in systematic reviews [7]. This study showed that these AI platforms have also been used to find research concerning other disciplines apart from orthopedics. Given the lack of orthopedic studies concerning ScholarGPT and the concomitant lack of papers to explore AI's ability to find orthopedic literature, we aimed to comparatively investigate ChatGPT’s and ScholarGPT’s ability to detect and outline publications related to orthopedic navigation.

## Materials and methods

Three orthopedic surgeons discussed finding a topic that would be given to ChatGPT and ScholarGPT for the detection and summarization of relevant literature. The topic that was chosen was augmented reality (AR)-based orthopedic navigation. The role of AR in preoperative planning and orthopedic navigation is well-documented [8-10]. This topic permitted further investigation of the potential of AR to enable anatomical accuracy in orthopedic surgery. The three surgeons formulated three queries to explore the two platforms’ ability to identify and summarize: 1) studies about AR navigation in general, 2) studies about AR navigation with a focus on anatomical accuracy in orthopedic interventions (to examine detection and summarization at a more advanced level), and 3) comparative studies regarding AR navigation (to achieve a different type of advanced level). The three queries (formulated after discussion) were: 1) Please list five peer-reviewed studies about augmented reality-based navigation in orthopedic surgery. Please summarize their results. 2) Please list five peer-reviewed studies about how well augmented reality-based navigation enabled anatomical accuracy in orthopedic surgery. Please summarize their results and do not repeat previously mentioned studies. 3) Please list five peer-reviewed studies that compared augmented reality-based navigation with another navigation technique in orthopedic surgery. Please summarize their results and do not repeat previously mentioned studies.

In case of repetition of previously mentioned studies, we did not ask again, because repetition was considered as the platform’s inflexibility in finding studies. The two accessed platforms were ChatGPT-4.0 turbo (https://chatgpt.com) and the ScholarGPT version of this platform (https://chatgpt.com/g/g-kZ0eYXlJe-scholar-gpt). These queries were submitted to both platforms on July 17, 2025. The three surgeons read the retrieved articles and independently evaluated how many of the five required papers were successfully detected and accurately summarized in each case. “Successful detection” means that the study’s title or at least one outcome was relevant to the query. “Accurate summary” was defined as the summary that contained true information about all the abstract’s outcomes relative to the query, including numerical data. The three surgeons qualitatively evaluated the responses for possible bias and discussed their final evaluation. In case of disagreement, the senior author decided.

## Results

The two platforms generated responses for all queries and provided a link for each study. These responses are presented in the Appendices. Their outline is the following.

ChatGPT’s response to the first query

Ma et al. found that AR led to significantly more accurate pedicle screw trajectories, lower cortical breach rate, and shorter screw placement time than free-hand surgery using computed tomography (CT) guidance [8]. Schwendner et al. (incorrectly cited as Ille et al.) showed that, with AR, 90.5% of pedicle screws were Gertzbein-Robbins Grade A or B, the screw revision rate was 1.7%, the average insertion time per screw was 6.5 min (with skin‑to‑incision-to‑reference imaging time 19 min), while there were no new postoperative neurological deficits [9]. Martínez et al. noted that AR resulted in the placement of acetabular implants in cadaveric hemipelves with a mean translational error of 3.79 mm and a mean angular error of 3.73°, while the workflow required no physical guides [10]. Kiarostami et al. showed that AR holds promise for guiding complex pelvic osteotomies [11]. Novice surgeons achieved periacetabular osteotomy accuracy in cadavers comparable to that of experienced surgeons using the free‑hand technique. Wang et al. found that AR led to reduced radiation exposure and procedure time of sacroiliac screw placement on cadaveric pelves, without loss in positional accuracy compared to conventional guidance (Table 1) [12].

**Table 1:** Summary of ChatGPT’s responses to the first query

Study	Main findings
Ma et al. [8]	Augmented reality led to significantly higher accuracy and shorter screw placement time than free-hand surgery using computed tomography guidance.
Schwendner et al. [9]	Augmented reality led to high accuracy of pedicle screws placement, without new postoperative neurological deficits.
Martínez et al. [10]	Augmented reality resulted in highly accurate placement of acetabular implants in cadaveric hemipelves.
Kiarostami et al. [11]	With augmented reality, novice surgeons achieved periacetabular osteotomy accuracy in cadavers comparable to experienced surgeons using the free‑hand technique.
Wang et al. [12]	Augmented reality led to less time of sacroiliac screw placement on cadavers, and less radiation, without loss in accuracy compared to conventional guidance.

ScholarGPT’s response to the first query

El-Hajj et al. compared AR, traditional navigation, and free-hand techniques in spinal deformity surgeries [13]. AR navigation significantly improved the accuracy of pedicle screw placement and reduced the operative time without increasing complications. Zhang et al. noted that AR navigation significantly improved the accuracy of femoral tunnel positioning during arthroscopic anterior cruciate ligament reconstruction, especially for novice surgeons, and shortened the learning curve in simulation environments [14]. In a cadaveric study, Molina et al. demonstrated that AR-based navigation improved the accuracy of pedicle screw insertion compared to traditional fluoroscopy [15]. Accuracy reached 94% with AR versus 85% with conventional methods, and AR eliminated radiation exposure.

The platform hallucinated the studies by "Wu et al." and "Khan et al.", mentioning the following findings: "In the meta-analysis of 14 trials by Wu et al., AR improved the accuracy of implant placement, shortened the operation time by approximately 17%, and showed the most benefit in minimally invasive surgery. Khan et al. showed that AR-based guidance in total knee replacement resulted in optimal alignment outcomes within 2° of planned angles." Surgeons reported reduced cognitive load and improved confidence (Table 2).

**Table 2:** Summary of ScholarGPT’s responses to the first query

Study	Main findings
El-Hajj et al. [13]	Compared to traditional navigation and free-hand techniques, augmented reality navigation significantly improved the accuracy and reduced the time of pedicle screw placement, without increasing complications.
Zhang et al. [14]	Augmented reality navigation significantly improved the accuracy of femoral tunnel positioning and shortened the learning curve of anterior cruciate ligament reconstruction.
Molina et al. [15]	Augmented reality navigation led to higher accuracy of pedicle screw insertion compared to traditional fluoroscopy and eliminated radiation exposure.
Wu et al.	Augmented reality improved the accuracy and reduced the time of implant placement, especially in minimally invasive surgery.
Khan et al.	Augmented reality-based guidance in total knee replacement resulted in high accuracy, while surgeons reported reduced cognitive load and improved confidence.

ChatGPT’s response to the second query

Wang et al. showed that when AR navigation was used for percutaneous sacroiliac screws insertion, the mean deviation at the entry point was 2.7 mm (at tip: 3.7 mm), the mean angular deviation was 2.9°, while no perforations occurred [12]. Hasegawa et al. found that AR‑based navigation in total hip arthroplasty led to mean angular navigation errors of 2.5° in inclination and 2.5° in anteversion, while pelvic posture variability modestly affected anteversion error [16]. Dennler et al. showed that AR-navigated sacral-alar-iliac screw insertion led to a perforation rate of 2.5%, which was significantly lower than the 60% rate associated with the free-hand technique [17]. AR navigation also resulted in improved axis deviation and screw length closer to optimal values. The meta-analysis by Youssef et al. showed that AR-assisted pedicle screw placement was associated with an overall anatomical accuracy (Gertzbein-Robbins grades 0 & 1) of 97.2%, a mean linear deviation between 1.3 and 5.99 mm, and an angular deviation between 1.6° and 5.88° [18]. Heining et al. found that ex vivo AR-guided pelvic screw placement was accurate, with the potential to reduce radiation versus conventional navigation (Table 3) [19].

**Table 3:** Summary of ChatGPT’s responses to the second query

Study	Main findings
Wang et al. [12]	Augmented reality navigation resulted in high accuracy of percutaneous sacroiliac screws insertion.
Hasegawa et al. [16]	Augmented reality navigation in total hip arthroplasty led to high accuracy, while pelvic posture variability modestly affected anteversion error.
Dennler et al. [17]	Augmented reality-navigated sacral-alar-iliac screw insertion led to a significantly lower perforation rate than the free-hand technique.
Youssef et al. [18]	Augmented reality-assisted pedicle screw placement was associated with high anatomical accuracy.
Heining et al. [19]	Ex-vivo augmented reality-guided pelvic screw placement was accurate, with potential to reduce radiation versus conventional navigation.

ScholarGPT’s response to the second query

Alekberov et al. evaluated how AR combined with three-dimensional (3D) printed anatomical models improved preoperative planning [20]. Surgeons achieved higher anatomical accuracy with AR, which aided in implant positioning and soft-tissue preservation. Baker et al. developed an AR platform for arthroscopic simulation [21]. By projecting real-time 3D patient anatomy into the surgeon’s field of view, AR enhances anatomical understanding and tool navigation, especially for trainees learning joint anatomy. Li et al. introduced an AR navigation system that overlaid 3D knee models onto the surgical field [22]. It significantly improved orientation and allowed for more accurate instrument tracking and anatomical landmark identification. In the study by De Rosa et al., surgeons used AR-enhanced planning to better visualize deformities and growth plate anatomy in a pediatric patient [23]. The study confirmed improved anatomical match between virtual models and intraoperative anatomy. Colcuc et al. compared AR and virtual reality platforms to CT-based planning for tibial plateau fractures [24]. AR showed higher anatomical fidelity and better visualization of posterior fragments, enabling improved screw trajectories and reduced intraoperative fluoroscopy (Table 4).

**Table 4:** Summary of ScholarGPT’s responses to the second query

Study	Main findings
Alekberov et al. [20]	Surgeons achieved higher anatomical accuracy with augmented reality, which aided in implant positioning and soft-tissue preservation.
Baker et al. [21]	Augmented reality enhanced anatomical understanding and tool navigation, especially for arthroscopy trainees learning joint anatomy.
Li et al. [22]	Augmented reality significantly improved knee surgery orientation and allowed for more accurate instrument tracking and anatomical landmark identification.
De Rosa et al. [23]	There was a satisfactory anatomical match between augmented reality models and the intraoperative anatomy of a pediatric patient.
Colcuc et al. [24]	Augmented reality showed higher anatomical fidelity and better visualization of posterior tibial plateau fracture fragments, compared to computed tomography-based planning.

ChatGPT’s response to the third query

Peh et al. found that AR navigation for thoracic and lumbar pedicle screws placement achieved an overall accuracy of 94% versus 88% achieved with fluoroscopy (a non-significant difference) [25]. In the thoracic region, the accuracy was 92% versus 83% respectively. AR eliminated intraoperative radiation exposure. Elmi-Terander et al. showed that AR-guided screw placement for spine fixation was 93.9% accurate versus 89.6% with the free-hand technique (a significant difference) [26]. The proportion of screws without any cortical breach was 63.4% versus 30.6%, respectively (a significant difference). There was no difference in procedure time, blood loss, or hospital stay. Elmi-Terander et al. found that AR-assisted pedicle screw insertion had an accuracy of 85% versus 64% with the free-hand technique (a significant difference) [27]. Perfect placements were achieved in 51% and 30% of cases, respectively, while breaches greater than 4 mm occurred in 2% of AR cases versus 25% of free-hand cases. In the study by Auloge et al., AR-guided percutaneous vertebroplasty and standard fluoroscopy exhibited a non-significant difference in trocar placement accuracy [28]. The procedure time was significantly longer with AR (642 sec versus 336 sec). Radiation dose and fluoroscopy time were approximately halved with AR (a significant difference). There were no complications. Chui et al. found that AR-guided tibial osteotomy had a lower preoperative planning time (4 min) versus conventional navigation (31 min) versus metal guide (75 min) [29]. The intraoperative times were 8.5 min, 31.5 min, and 10.5 min, respectively. AR also resulted in higher accuracy (Table 5).

**Table 5:** Summary of ChatGPT’s responses to the third query

Study	Main findings
Peh et al. [25]	Augmented reality navigation for pedicle screws placement eliminated intraoperative radiation exposure and achieved a non-significantly different accuracy compared to fluoroscopy.
Elmi-Terander et al. [26]	Augmented reality-guided screws placement for spine fixation was significantly more accurate compared to the free-hand technique. There was no difference in procedure time, blood loss, or hospital stay.
Elmi-Terander et al. [27]	Augmented reality-assisted pedicle screw insertion had a significantly higher accuracy than the free-hand technique.
Auloge et al. [28]	Augmented reality-guided vertebroplasty and standard fluoroscopy exhibited a non-significant difference in trocar placement accuracy. Augmented reality resulted in a significantly longer procedure time and a significantly lower radiation dose and fluoroscopy time.
Chui et al. [29]	Augmented reality-guided tibial osteotomy had a lower preoperative planning time than conventional navigation versus metal guide. Augmented reality resulted in higher accuracy than its comparators.

ScholarGPT’s response to the third query

Elmi-Terander et al. showed that AR navigation achieved 94.1% Gertzbein-Robbins Grade A accuracy, significantly outperforming traditional fluoroscopy-assisted free-hand techniques [30]. AR also enabled continuous visualization without requiring surgeons to shift their visual focus.

The platform hallucinated the studies by "Guha et al.", "Friedrich et al.", "Ma et al.", and "Zhou et al.", mentioning the following findings: "Guha et al. found that AR navigation demonstrated accuracy comparable or superior to traditional image-guided navigation in pedicle screw placement. Participants using AR also reported a reduced mental workload and improved 3D anatomical perception. Friedrich et al. compared AR-assisted distal locking of intramedullary nails to conventional fluoroscopy for long bone fractures. AR reduced radiation exposure by 71%, shortened procedure times by 20%, and achieved similar or higher locking accuracy. In a comparison of AR and robotic navigation for lumbar pedicle screw placement, Ma et al. found that while robotic systems offered slightly higher placement accuracy, AR was significantly faster, less expensive, and did not require extensive preoperative setup. Zhou et al. evaluated AR versus CT-based navigation for distal radius osteotomies. AR-guided procedures had shorter operative times, higher anatomical congruence, and easier intraoperative adaptability for varying patient anatomies" (Table 6).

**Table 6:** Summary of ScholarGPT’s responses to the third query

Study	Main findings
Guha et al.	Augmented reality navigation demonstrated accuracy comparable or superior to traditional image-guided navigation in pedicle screw placement.
Elmi-Terander et al. [30]	Augmented reality navigation achieved high accuracy, significantly outperforming traditional fluoroscopy-assisted free-hand techniques.
Friedrich et al.	Augmented reality resulted in similar or higher accuracy of distal locking of intramedullary nails compared to conventional fluoroscopy.
Ma et al.	While robotic systems offered slightly higher pedicle screw placement accuracy, augmented reality was significantly faster, less expensive, and did not require extensive preoperative setup.
Zhou et al.	Compared to computed tomography-based navigation, augmented reality-guided distal radius osteotomies had shorter operative times, higher anatomical congruence, and easier intraoperative adaptability for varying patient anatomies.

## Discussion

Regarding the first query, ChatGPT correctly identified all required studies (100%) [8-12] and accurately summarized two [8,9] studies (40%). In the study by Martínez et al., the platform omitted to mention that, compared to previous phantom-based experiments, the AR errors were significantly greater, probably due to the anatomical complexity of cadaveric specimens [10]. Regarding the study by Kiarostami et al., ChatGPT failed to note that AR guidance did not influence the expert surgeon’s performance in terms of the mean differences between the planned and executed starting points [11]. The study by Wang et al. was also inaccurately summarized [12]. Despite ChatGPT’s statement, this paper did not compare AR with conventional navigation, nor did it evaluate radiation exposure or procedure duration.

ScholarGPT correctly identified three [13-15] out of the five required studies (60%), but there was no accurately summarized study (0%). The platform hallucinated two studies by “Wu et al.” and “Khan et al.”. The paper by El-Hajj et al. [13] was correctly detected, but ScholarGPT hallucinated the existence of research findings (this paper concerned a study protocol). The publication by Zhang et al. was a review about all navigation technologies used in anterior cruciate ligament reconstruction, without specific outcomes about AR in the abstract (despite otherwise stated) [14]. Of note, in these two cases [13,14], the false generated outcomes were in favor of AR. Molina et al. [15] found that AR-guided screw insertion accuracy was, in some cases, non-inferior to the accuracy achieved by other methods. However, this finding was absent from ScholarGPT’s generated text.

Regarding the second query, ChatGPT correctly identified five [12,16-19] studies (100%) and accurately summarized three [12,16,17] studies (60%). The paper by Wang et al. was again listed after the first query, despite the relative instruction [12]. However, in contrast with the previous query, the summarization of the study was adequate. The summary of the systematic review by Youssef et al. [18] lacked information about breach percentages, while the real percentage of clinically accurate screws was not 97.2%, but 93.1%. The outline of the paper by Heining et al. did not contain the findings concerning the success rate for entry point navigation, translational and rotational deviation of drill pathways, and pelvic screw perforation scores [19].

On the other hand, ScholarGPT correctly detected two [20,21] out of the five required studies (40%) and accurately summarized one [20] of them (20%). The platform listed two studies about virtual reality [23,24] (although the summaries generated were about AR) and one study about augmented virtuality [22]. Also, Baker et al. [21], in contrast with ScholarGPT’s statement, did not develop an AR platform for arthroscopic simulation, but just published a review article about AR in orthopedic surgery and education. In all these cases, only positive outcomes of AR navigation were mentioned [21-24].

Regarding the third query, ChatGPT correctly identified five [25-29] studies (100%) and accurately summarized three [26-28] of them (60%). In the paper by Peh et al., the platform failed to point out that there was no significant difference between AR and fluoroscopy in the median time for K-wire placement [25]. Of note, in the study by Chui et al., ChatGPT stated that AR enabled higher accuracy than conventional navigation and metal guide [29]. However, the authors found that AR exhibited lower accuracy than the other two methods.

On the other hand, ScholarGPT did not correctly identify any studies (0%) (Table 7). From the five retrieved studies, only that by Elmi-Terander et al. exists, yet it was not correctly identified, because it was not comparative [30]. Of note, in this paper, ScholarGPT hallucinated that AR was significantly superior to traditional fluoroscopy.

**Table 7:** Summary of ChatGPT’s and ScholarGPT’s performances

Query	ChatGPT’s successful detection	ChatGPT’s accuracy	ScholarGPT’s successful detection	ScholarGPT’s accuracy
Studies about augmented reality-based navigation	100%	40%	60%	0%
Studies about how well augmented reality-based navigation enabled anatomical accuracy	100%	60%	40%	20%
Studies which compared augmented reality with another navigation technique	100%	60%	0%	0%

Overall, ChatGPT’s performance in identifying the required studies was excellent across all three queries. However, its success in accurately summarizing these studies just surpassed 50%. Given that the second query was more complex than the first one, it seems that ChatGPT’s performance was not influenced by this complexity. Also, ChatGPT showed bias toward reporting data in favor of AR. There was only one case where ChatGPT mentioned a disadvantage of AR [28], while in one case [10], the platform omitted outcomes significantly inferior to comparators. In two cases [18,29], the results after AR navigation were presented better than the real ones, and in two more cases [11,25], the platform did not mention neutral AR outcomes. In one case, ChatGPT hallucinated AR's superiority to conventional navigation, though the study was not comparative [12]. Furthermore, in one case, ChatGPT presented the second author of the study as the first [9]. On the other hand, ScholarGPT’s performance was consistently poor across all three queries. It successfully identified only one-third of the required studies (33.3%) and accurately summarized only 1 out of the 15 required studies (6.7%). Of note, it hallucinated the existence of six studies, while it generated false findings in seven cases [13,14,21-24,30], always in favor of AR. Also, in one case, ScholarGPT mentioned only positive outcomes about AR, while omitting to point out a neutral finding [15]. Thus, this platform also exhibited bias in favor of AR.

Our study has limitations. ChatGPT’s and ScholarGPT’s evaluation included just percentages of correct detection and accurate summarization. The existence of a specific evaluation rubric in the literature could generate more robust data in future studies. Also, the two platforms were asked on a specific day and time. If they are tested in other scientific disciplines, or at different times, or with different questions, they may perform differently. It is expected that the ongoing AI evolution may reinforce ChatGPT’s and ScholarGPT’s ability to play a more remarkable role in orthopedic research.

## Conclusions

ChatGPT performed excellently in identifying studies on AR navigation in orthopedics and related surgical anatomy, while its ability to accurately summarize these studies was moderate. In contrast, ScholarGPT’s performance was consistently poor. Both platforms exhibited considerable bias in favor of AR navigation. Our findings indicate that these two models are not yet able to provide reliable data from the literature about orthopedic navigation. Ongoing AI development may essentially reinforce these platforms’ support to orthopedic researchers.

## Appendices

First query: Please list five peer-reviewed studies about augmented reality-based navigation in orthopedic surgery. Please summarize their results.

Second query: Please list five peer-reviewed studies about how well augmented reality-based navigation enabled anatomical accuracy in orthopedic surgery. Please summarize their results and do not repeat previously mentioned studies.

Third query: Please list five peer-reviewed studies which compared augmented reality-based navigation with another navigation technique in orthopedic surgery. Please summarize their results and do not repeat previously mentioned studies.

ChatGPT's responses to the first query

1. Ma et al. (2025) - Randomized multicenter clinical trial on spinal AR navigation for pedicle screw placement

Design: 150‑patient RCT comparing AR‐guided screw insertion vs. conventional free‑hand in lumbar spine surgeries.

Results:

98% accurate screw trajectories in the AR group vs. 92.7% in manual control (statistically significant)

Lower rate of cortical breaches in AR (17%) vs. manual (70.6%)

Higher grade-A facet joint penetration: 97.2% in AR, 83.3% in manual

Screw placement time per screw is significantly shorter in AR (≈ 16.3 min) vs. manual (≈ 30.3 min)

Conclusion: AR guidance notably improves accuracy, safety, and efficiency relative to traditional manual methods in lumbar spine surgery

2. Ille et al. / European Spine Journal (2023) - Clinical evaluation of AR-supported navigation system

Design: Clinical study (~20 patients) using AR glasses integrated with camera-based navigation for pedicle screw placement across indications (degenerative, trauma, infection, tumor).

Results: 90.5% of screws graded A/B

Screw revision rate 1.7%

No new postoperative neurological deficits

Average insertion time per screw ~6.5 min (skin‑to‑incision-to‑reference imaging time ≈ 19 min)

Conclusion: AR integration is feasible and precise in real-world spine surgeries, with low revision rates and preserved safety

3. Orozco‑Martínez et al. (2025) - Cadaveric study for patient‑specific implant placement in orthopedic oncology

Design: Ten cadaveric hemipelves with custom acetabular implants, guided via HoloLens and marker-based AR feedback.

Results:

Mean translational error ~3.79 mm (SD ± 2.09 mm)

Mean angular error ~3.73° (SD ± 2.59°)

Placement workflow required no physical guides and used CT overlap and color-coded feedback

Conclusion: AR allows feasible and sufficiently accurate placement of patient-specific implants in anatomically complex cadaveric pelvis models, suggesting readiness for surgical translation

4. Kiarostami et al. (Applied Sciences, 2021) - Feasibility of AR-guided periacetabular osteotomy (PAO) in cadavers

Design: Two cadaver PAO operations using off‑the‑shelf OST-HMD (HoloLens), marker‑less inside‑out tracking and AR overlay for planning and fragment reduction.

Results:

Novice surgeons achieved osteotomy accuracy comparable to experienced PAO surgeons doing free‑hand

Demonstrated feasibility of full pipeline from 3D planning to execution using AR

Conclusion: Marker‑less AR navigation holds promise for guiding complex pelvic osteotomies, even by less experienced operators

5. Wang et al. / in vitro cadaveric pelvic fracture navigation (J Med Internet Res review, 2023)

Design: Cadaveric pelvis experiments for percutaneous sacroiliac screw insertion using AR overlay vs. standard C‑arm fluoroscopy.

Results:

AR guidance feasible and accurate in small pelvis models

Reduced radiation exposure and procedure time

No loss in positional accuracy vs. conventional guidance

Conclusion: AR is a potentially valuable adjunct in minimally invasive pelvic fracture fixation, reducing time and radiation without compromising accuracy

ChatGPT's responses to the second query

1. Precision insertion of percutaneous sacroiliac screws using AR navigation (International Orthopaedics, 2015)

Model: Cadaver pelvises (n = 6 cadavers, 12 screws)

Accuracy results:

No perforations occurred

Mean deviation at entry point: 2.7 ± 1.2 mm; at tip: 3.7 ± 1.1 mm

Angular deviation: 2.9° ± 1.1°

Conclusion: The AR-guided system enabled highly accurate screw alignment relative to the planned trajectory, with negligible breaches.

Angular deviation: 2.9° ± 1.1°

2. Accuracy of AR‑based CT navigation in total hip arthroplasty (THA) (Journal of Orthopaedic Surgery and Research, 2023)

Context: 65 patients undergoing primary cementless THA with AR overlay

Results:

Mean angular navigation errors: 2.5° ± 1.7° in inclination; 2.5° ± 2.2° in anteversion

Pelvic posture variability modestly affected anteversion error

Conclusion: AR navigation achieved clinically acceptable cup placement accuracy, improving over freehand approaches.

3. AR-navigated sacral-alar-iliac (SAI) screw insertion (lab model, 2021)

Model: Pelvic sawbone trials, 80 screw trajectories by two operators

Findings:

Perforations: 2.5% with AR versus 60% in freehand (P < .05)

Improved alignment metrics (axis deviation, screw length) nearer to optimal values

Conclusion: AR significantly enhanced anatomical precision in pilot drilling for S2‑AI screw corridors under controlled conditions.

4. Meta‑analysis of AR-assisted pedicle screw placement (European Spine Journal, 2024)

Scope: Systematic review covering 8 studies, 1,259 screws

Results:

Overall anatomical accuracy (Gertzbein-Robbins grades 0 & 1): 97.2% (CI 96.2-98.1%)

Mean linear deviation: 1.3-5.99 mm; angular deviation: 1.6°-5.88°

Conclusion: AR navigation yields high anatomical precision across various spine settings, largely matching or surpassing conventional navigation.

5. AR-guided pelvic screw placement feasibility study (Patient Safety in Surgery, 2023)

Model: Ex‑vivo (cadaver-like) experiment navigating 12 difficult pelvic screw pathways

Observations:

Feasible and accurate screw placement under clinical-like conditions

Enabled true 3D anatomical alignment (transparency mode) with potential to reduce radiation versus conventional navigation

Conclusion: AR navigation can achieve anatomical accuracy comparable to other navigational systems while providing immersive guidance.

ChatGPT's responses to the third query

1. ARSN vs. Fluoroscopy-Guided MIS Pedicle Screw Placement (cadaveric lab study)

Setting: Four cadaver spines; 136 thoracic/lumbar screws from ARSN vs standard fluoroscopy-guided minimally invasive technique

Results:

Overall accuracy: 94% (ARSN) vs 88% (fluoroscopy), difference did not reach statistical significance

Thoracic region: 92% (ARSN) vs 83% (fluoroscopy) accuracy

ARSN eliminated intraoperative radiation exposure for the surgeon

Conclusion: ARSN was at least as accurate as fluoroscopy-guided MIS navigation, with the advantage of zero surgeon radiation exposure

2. ARSN vs Free-Hand Screw Placement (matched-control clinical study)

Setting: Twenty patients with ARSN guidance matched to 20 retrospective free-hand cases; total of ~262 vs ~288 screws

Results:

Clinically accurate screws (Gertzbein grades 0 & 1): 93.9% (ARSN) vs 89.6% (free-hand), p < 0.05

No cortical breach screws: 63.4% (ARSN) vs 30.6% (free-hand), p < 0.0001

No difference in procedure time, blood loss, or hospital stay

Conclusion: ARSN significantly improved anatomical accuracy over free-hand technique while maintaining similar operative metrics

3. ARSN vs Free-Hand Screw Placement in Thoracic Spine (paired cadaveric study)

Setting: 94 thoracic pedicle screws in four cadavers; ARSN applied on one side, free-hand on the contralateral side

Results:

Accuracy: 85% (ARSN) vs 64% (free-hand), p < 0.05

Perfect placements: 51% (ARSN) vs 30% (free-hand)

Breaches >4 mm: 2% (ARSN) vs 25% (free-hand)

Conclusion: ARSN yielded significantly higher thoracic pedicle accuracy than free-hand placement, with fewer major breaches

4. AR/AI-Guided Percutaneous Vertebroplasty vs Standard Fluoroscopy (randomized clinical trial)

Setting: 10 patients using AR/AI guidance (trocar insertion) vs 10 controls under standard fluoroscopy

Results:

Accuracy (trajectory deviation): ~1.0-1.9 mm RMS in both groups (no significant difference)

Procedure time: longer for AR/AI group (~642 s vs ~336 s, p = 0.001)

Radiation dose and fluoroscopy time roughly halved in AR/AI group (p < 0.05)

No complications reported

Conclusion: AR/AI guidance matched standard fluoroscopy in accuracy while significantly reducing radiation exposure, albeit with longer procedural time

5. AR Navigation vs Conventional Navigation & Surgical Guide in Knee Osteotomy (sawbone trials)

Setting: Sawbone HTO (high tibial osteotomy) cutting guided by three methods: AR navigation system, conventional navigation system, and metal 3D printed guide

Results:

Pre-op planning time: AR most rapid (~4 min) vs conventional (~31 min) vs printed guide (~75 min)

Intra-op cutting time: AR fastest (~8.5 min) vs conventional (~31.5 min) and guide (~10.5 min)

Accuracy: AR > conventional nav > metal 3D guide (angle deviation lowest for AR)

Conclusion: AR navigation enabled faster planning and execution and delivered superior cutting accuracy relative to both conventional navigation and static guides

ScholarGPT's responses to the first query

1. El-Hajj, V.G., Charalampidis, A., Fell, D., & Edström, E. (2025).

Title: The SPInal NAVigation (SPINAV) Trial - Comparison of Augmented Reality Surgical Navigation, Conventional Image-Guided Navigation, and Free-Hand Techniques in Spine Surgery

Journal: BMC Musculoskeletal Disorders

Summary: This prospective trial compared AR, traditional navigation, and free-hand techniques in spinal deformity surgeries. AR navigation significantly improved pedicle screw placement accuracy and reduced operative time without increasing complications. Surgeons reported better spatial awareness using AR headsets.

2. Zhang, Z., Meng, B., Li, W., & Cao, J. (2025).

Title: The Role of Navigation Technology in Anterior Cruciate Ligament Reconstruction Bone Tunnel Positioning 

Journal: Journal of Robotic Surgery

Summary: This study analyzed AR-assisted systems in arthroscopic ACL reconstruction. AR navigation significantly improved the accuracy of femoral tunnel positioning, especially for novice surgeons, and shortened the learning curve in surgical simulation environments.

3. Molina, C.A., Theodore, N., & Mure, A. (2020).

Title: Augmented Reality-Assisted Pedicle Screw Insertion: A Cadaveric Study

Journal: Spine

Summary: In this cadaver study, AR-based navigation improved the accuracy of pedicle screw insertion compared to traditional fluoroscopy. Accuracy reached 94% with AR vs. 85% with conventional methods, and AR reduced radiation exposure to zero.

4. Wu, A.M., et al. (2022).

Title: Augmented Reality in Orthopedic Surgery is a Leap Forward: A Meta-Analysis of Accuracy and Efficiency

Journal: Journal of Orthopaedic Surgery and Research

Summary: A meta-analysis of 14 trials involving AR navigation across orthopedic procedures. AR improved placement accuracy (especially for spinal and hip implants), shortened operation time by ~17%, and showed the most benefit in minimally invasive surgery.

5. Khan, R., et al. (2021).

Title: Real-Time AR Headset Navigation for Total Knee Arthroplasty: A First-in-Human Pilot

Journal: Journal of Arthroplasty

Summary: In this pilot clinical trial using Microsoft HoloLens, AR-based guidance in total knee replacement resulted in optimal alignment outcomes within 2° of planned angles. Surgeons also reported reduced cognitive load and improved confidence.

ScholarGPT's responses to the second query

1. Alekberov, R.R., Zhedrinskaya, M.V., Korzan, A.A., et al. (2022).

Title: The Use of Augmented Reality and 3D Printing Technologies in Planning and Conducting Surgical Intervention in Orthopedics

Journal: Transactions on Engineering and Technology

Summary: This study evaluated how AR combined with 3D printed anatomical models improved surgical precision in preoperative planning for joint surgeries. Surgeons achieved higher anatomical accuracy when visualizing DICOM scans in AR, aiding in implant positioning and soft-tissue preservation.

2. Baker, K., Fryberger, T., & Ponce, A. (2015).

Title: The Emergence of Augmented Reality in Orthopaedic Surgery and Education

Journal: The Orthopaedic Journal at Harvard Medical School

Summary: This paper described the development of an AR platform for arthroscopic simulation. By projecting real-time 3D patient anatomy into the field of view, AR enhanced anatomical understanding and tool navigation, especially for trainees learning joint anatomy.

3. Li, J.M., Bardana, D.D., & Stewart, A.J. (2011).

Title: Augmented Virtuality for Arthroscopic Knee Surgery

Conference: Medical Image Computing and Computer-Assisted Intervention (MICCAI)

Summary: The study introduced an AR navigation system overlaying 3D knee models onto the surgical field. It significantly improved orientation within complex joint spaces and allowed for more accurate instrument tracking and anatomical landmark identification.

4. De Rosa, A., Alessandri, G., Pignatelli, E., et al. (2024).

Title: Exploring Virtual Reality Surgical Planning Applications in Paediatric Orthopaedics: A Preliminary Case Study

Conference: International Conference on Extended Reality

Summary: In a pediatric orthopedic case, surgeons used AR-enhanced planning to better visualize deformities and growth plate anatomy. The study confirmed improved anatomical matching between virtual models and intraoperative anatomy, helping avoid critical structures.

5. Colcuc, C., Miersbach, M., Cienfuegos, M., et al. (2024).

Title: Comparison of Virtual Reality and Computed Tomography in the Preoperative Planning of Complex Tibial Plateau Fractures

Journal: Archives of Orthopaedic and Trauma Surgery

Summary: This study compared AR/VR platforms to CT-based planning for tibial plateau fractures. AR showed higher anatomical fidelity and better visualization of posterior fragments, enabling improved screw trajectories and reduced intraoperative fluoroscopy.

ScholarGPT's responses to the third query

1. Guha, D., et al. (2020).

Title: Augmented Reality-Assisted Spine Surgery: Evaluation of Performance in a Simulated Setting Compared to Conventional Image-Guided Navigation

Journal: Spine

Summary: In this simulated spine surgery study, AR navigation demonstrated comparable or better accuracy in pedicle screw placement versus traditional image-guided navigation. Participants using AR also had reduced mental workload and reported improved 3D anatomical perception.

2. Elmi-Terander, A., Nachabe, R., Skulason, H., et al. (2018).

Title: Feasibility and Accuracy of Thoracolumbar Minimally Invasive Pedicle Screw Placement With Augmented Reality Navigation Technology

Journal: Spine

Summary: In a clinical setting, AR navigation achieved an impressive 94.1% Gertzbein-Robbins Grade A accuracy, significantly outperforming traditional fluoroscopy-assisted free-hand techniques. The system also enabled continuous visualization without the need to shift visual focus.

3. Friedrich, R., et al. (2023).

Title: Clinical Application of Augmented Reality-Assisted Navigation in Intramedullary Nailing: A Comparative Study With Fluoroscopy

Journal: Journal of Orthopaedic Surgery and Research

Summary: Compared AR-assisted distal locking of intramedullary nails with conventional fluoroscopy in long bone fractures. AR reduced radiation exposure by 71%, shortened procedure times by 20%, and achieved similar or higher locking accuracy.

4. Ma, L., Zhao, Z., Chen, H., et al. (2021).

Title: A Comparative Study of Augmented Reality Navigation vs. Robot-Assisted Navigation in Spine Surgery

Journal: Orthopaedic Surgery

Summary: This head-to-head study compared AR and robotic navigation for lumbar pedicle screw placement. While robotic systems had slightly higher placement accuracy, AR was significantly faster, less expensive, and did not require extensive preoperative setup.

5. Zhou, L., et al. (2022).

Title: Comparative Effectiveness of Augmented Reality-Guided Versus Conventional Navigation in Corrective Osteotomy of the Distal Radius

Journal: Journal of Hand Surgery Global Online

Summary: This study evaluated AR vs. CT-based navigation for distal radius osteotomies. AR-guided procedures had shorter operative times, higher anatomical congruence (measured via postoperative CT), and easier intraoperative adaptability for varying patient anatomies.
